# Clinical Features and Imaging Findings of Myelin Oligodendrocyte Glycoprotein-IgG-Associated Disorder (MOGAD)

**DOI:** 10.3389/fnagi.2022.850743

**Published:** 2022-03-15

**Authors:** Yunjie Li, Xia Liu, Jingxuan Wang, Chao Pan, Zhouping Tang

**Affiliations:** Department of Neurology, Tongji Hospital, Tongji Medical College, Huazhong University of Science and Technology, Wuhan, China

**Keywords:** MOGAD, optic neuritis, myelitis, MOG-IgG, clinical review

## Abstract

Myelin oligodendrocyte glycoprotein-IgG-associated disorder (MOGAD) is a nervous system (NS) demyelination disease and a newly recognized distinct disease complicated with various diseases or symptoms; however, MOGAD was once considered a subset of neuromyelitis optica spectrum disorder (NMOSD). The detection of MOG-IgG has been greatly improved by the cell-based assay test method. In one study, 31% of NMOSD patients with negative aquaporin-4 (AQP-4) antibody were MOG-IgG positive. MOGAD occurs in approximately the fourth decade of a person’s life without a markedly female predominance. Usually, optic neuritis (ON), myelitis or acute disseminated encephalomyelitis (ADEM) encephalitis are the typical symptoms of MOGAD. MOG-IgG have been found in patients with peripheral neuropathy, teratoma, COVID-19 pneumonia, etc. Some studies have revealed the presence of brainstem lesions, encephalopathy or cortical encephalitis. Attention should be given to screening patients with atypical symptoms. Compared to NMOSD, MOGAD generally responds well to immunotherapy and has a good functional prognosis. Approximately 44-83% of patients undergo relapsing episodes within 8 months, which mostly involve the optic nerve, and persistently observed MOG-IgG and severe clinical performance may indicate a polyphasic course of illness. Currently, there is a lack of clinical randomized controlled trials on the treatment and prognosis of MOGAD. The purpose of this review is to discuss the clinical manifestations, imaging features, outcomes and prognosis of MOGAD.

## Introduction

Myelin oligodendrocyte glycoprotein (MOG) is widely present on the surface of oligodendrocytes and the myelin sheath of the nervous system, and its role may be similar to that of a cell adhesion molecule, maintaining the stability of the surface of oligodendrocytes and regulating the complement response (Bernard et al., 1997; Johns and Bernard, 1999). MOG-IgG can lead to ON, myelitis, and ADEM and are currently associated with anti-n-methyl-d-aspartate (NMADA) antibody encephalitis, teratoma, COVID-19, etc. (Fujimori et al., 2021; Peters et al., 2021; Wildemann et al., 2021). With the popularization of cell-based assay detection methods, MOGAD has been separated from NMOSD (Wingerchuk et al., 2015; Thompson et al., 2018). MOGAD is a demyelinating disease of the central nervous system (CNS). Typical symptoms of MOGAD include ON and myelitis, which overlap with multiple sclerosis (MS) and NMOSD (Carandini et al., 2021). Although the specific pathophysiologic mechanism remains inconclusive, MOGAD usually manifests as direct demyelinating pathological changes that are similar to MS, unlike NMOSD, in which astrocytes are first damaged and then demyelinated (Salama et al., 2019; Mader et al., 2020). Additionally, there is no unified standard regarding the clinical manifestations and magnetic resonance imaging (MRI) characteristics of MOGAD. Many atypical symptoms or complications have been reported (Fujimori et al., 2021; Peters et al., 2021; Wildemann et al., 2021). This paper discusses the typical symptoms and atypical symptoms of MOGAD through literature retrieval to improve the ability to identify potential patients.

### Optic Neuritis

#### Epidemic

Optic neuritis (ON) is the most common symptom of MOGAD in adults, occurring in approximately 54-61% of patients (Biotti et al., 2017; Hacohen et al., 2017; Cobo-Calvo et al., 2018; Carandini et al., 2021; Kunchok et al., 2021b). Miller et al. (2020) prospectively observed 65 patients diagnosed with acute ON within 1 year, 14% of whom were MOG-IgG positive. Akaishi et al. (2019) found that out of 166 MOGAD episodes in 85 patients, 67.5% were ON (bilateral neuritis was 18.7%). Compared with patients with MS or NMOSD, those with MOGAD present with isolated ON without additional CNS lesions (Wingerchuk et al., 2015). Netravathi et al. (2020) analysis of 263 CNS demyelination episodes in 93 MOGAD patients showed that 121 (45.8%) were ON. Other studies have reported similar results (Cobo-Calvo et al., 2017, 2021; Li et al., 2021; Rempe et al., 2021). Significantly, retrospective analysis results of Kitley et al. (2014) showed that among 9 MOGAD patients, none were diagnosed with ON alone, and 4 patients were diagnosed with ON plus myelitis simultaneously or successively. However, that study was very small and lacked sufficient statistical power.

#### Symptom Features

Bilateral involvement of the ON is usually present, but sometimes it may be unilateral ON (Akaishi et al., 2019; Rempe et al., 2021). Rempe’s study showed that compared with NMOSD patients, MOGAD patients with ON were more prone to bilateral optic nerve involvement (6/11 [54.5%] vs. 6/43 [13.9%]; *p* = 0.009) (Rempe et al., 2021). Some scholars have concluded that the anterior optic nerve is significantly more likely to be involved in MOGAD patients, which is different from AQP-4 antibody-positive patients (Kitley et al., 2014; Ducloyer et al., 2020). As we searched, it became clear that MOGAD patients usually have longitudinally bilateral optic nerve swelling, and abnormal signals around sheath or adipose tissue were occasionally found on imaging (Deneve et al., 2019; Ducloyer et al., 2020; Shor et al., 2021). Meta-analysis results of Carandini et al. (2021) showed that retrobulbar ON (*p* = 0.0006) is usually caused by MOG-IgG, accompanied by optic papilloedema (*p* < 0.00001). Gao et al. (2021) recently used ophthalmological indicators (fractional anisotropy, primary visual cortex volume, visual acuity) to analyze the structural and functional changes after ON in MOGAD patients, and the results showed that the AQP-4 group exhibited lower indicator values than the healthy control group, but there was no difference in the values between the MOGAD group and the control group, which may be the reason why MOGAD patients usually had a better prognosis. These results indicated that optic nerve damage in MOGAD patients was less severe than that in AQP-4 antibody-positive patients (Table 1).

**TABLE 1:** Optic neuritis comparison between MS, NMOSD, and MOGAD.

	MOGAD	NMOSD	MS
Age; Hoftberger et al., 2015; Jurynczyk et al., 2015; Wynford-Thomas et al., 2019; Gospe et al., 2021	40s	40s	30s
Female:male; Orton et al., 2006; Jarius et al., 2012; Jurynczyk et al., 2017	1.3:1	9:01	3:01
Clinical features; Jarius et al., 2012; Kitley et al., 2014; Jurynczyk et al., 2015; Narayan et al., 2018	usually bilateral with severe damage	usually bilateral with severe damage	unilateral is common
	longitudinally involvement is observed	extent of optic neuritis is longitudinal	short-segment is common
	unilateral is rare		
MRI features; Kitley et al., 2014; Carandini et al., 2021	optic nerve thickness and hyperintense on T2 sequence	optic nerve thickness and hyperintense on T2 sequence	optic nerve thickness and hyperintense on T2 sequence
	edema of the optic nerve sheath and inflammatory swelling of surrounding tissues		
Location; Kitley et al., 2014; Carandini et al., 2021	anterior part of the optic nerve and the retrobulbar	canalicular and intracranial tracts of the ON	anywhere in the optic nerve tract
	rarely involving the optic chiasma	can involve the optic chiasm	intra-orbital segment
		posterior optic pathway with chiasmal and optic tract involvement	
Disease course; Wynford-Thomas et al., 2019; Akaishi et al., 2021	monophasic or relapsing	relapsing	relapsing or progressive
Outcome; Cellina et al., 2019; Carandini et al., 2021	good recovery	limited recovery	good recovery

*MOGAD, myelin oligodendrocyte glycoprotein-IgG-associated disorder; NMOSD, neuromyelitis optica spectrum disorder; MS, multiple sclerosis.*

#### Magnetic Resonance Imaging Features

In general, MRI of the optic nerve in MOGAD patients shows hyperintensity on T2-weighted images and significant enhancement after gadolinium administration. Abnormal optic nerve signals are located in the anterior part of the optic nerve and the retrobulbar region, rarely involving the optic chiasma (Carandini et al., 2021). The extent of ON is usually more than half of the optic nerve length (Shor et al., 2021). Sometimes, imaging can show edema of the optic nerve sheath and inflammatory swelling of surrounding tissues (Kitley et al., 2014), indicating extensive optic nerve damage in MOGAD patients. During the follow-up period, optic nerve atrophy and optic nerve head thickness are also observed, which differ from the visual outcome (Deschamps et al., 2018; Shor et al., 2020).

#### Outcome and Prognosis

Previous results have reported that demyelinating changes occur directly in MOGAD patients, so visual loss is usually more severe and recovery is better than in AQP-4 antibody-positive patients (Cobo-Calvo et al., 2018; Mader et al., 2020; Gao et al., 2021). The clinical manifestations of adult MOGAD patients and AQP-4 antibody-positive patients have been compared, and the results showed that MOGAD patients had a lower probability of visual acuity (VA) of 20/100 (HR 0.23, 95% CI 0.07–0.72, *p* = 0.012) and severe initial VA loss (HR 1.52, 95% CI 1.12–2.05, *p* = 0.007) (Cobo-Calvo et al., 2018). Ramanathan et al. (2016) compared the differences among MS, NMOSD, and MOGAD patients in the manifestations of ON, and the results showed that MOGAD patients experienced severe visual impairment in the acute phase, with some patients even reaching a maximum visual functional system score (VFSS) of six. However, during the follow-up period, VA was obviously improved in most patients, and no patient had a maximum VFSS of 6. Jarius’s multicenter experiment produced similar results (Jarius et al., 2016). Kitley et al. (2014) used the expanded disability status scale (EDSS) score difference to evaluate the recovery of MOGAD patients and showed that compared with AQP-4 antibody-positive patients, MOGAD patients’ EDSS scores decreased significantly.

#### Relapse

Similar to NMOSD patients, at least 50% of MOGAD patients had a strong tendency to relapse (Jurynczyk et al., 2017; Kortvelyessy et al., 2017; Chaudhuri et al., 2021). ON is the most common clinical manifestation in patients with recurrent MOGAD (Cobo-Calvo et al., 2017; Wynford-Thomas et al., 2019; Chen et al., 2020). Specific indicators for relapse are not clear. The results of Jurynczyk et al. (2017) and Netravathi et al. (2020) showed that MOGAD patients with the first symptom of ON were more likely to relapse (Jurynczyk et al., 2017; Netravathi et al., 2020; Xu et al., 2021). Persistent high titers of MOG-IgG are a risk factor for recurrent events (Hennes et al., 2017; Lopez-Chiriboga et al., 2018). The Cobo-Calvo’s study showed that the MOG-IgG titers were significantly higher at relapse than remission (*P* = 0.009) (Cobo-Calvo et al., 2018). Some studies have also shown that a shorter immunotherapy cycle (≤ 3 months) is more likely to lead to recurrence (Sato et al., 2014; Cobo-Calvo et al., 2017; Jurynczyk et al., 2017). The results of Cobo-Calvo’s study showed that women and MOGAD patients treated with MS-DMT were at higher risk of relapse (Cobo-Calvo et al., 2021). The results of Cobo-Calvo et al. (2018) showed that there was no significant difference in the ratio of 20/100 VA recovery between patients with monophasic disease or recurrence. This suggests that ON is still recovering well after recurrence.

### Myelitis

#### Epidemic

In general, myelitis is a common manifestation in adults with MOGAD, occurring either alone or in conjunction with ON (Mader et al., 2018; Nagabushana et al., 2019; Suzuki et al., 2019). Akaishi et al. (2019) summarized 85 MOGAD patients and found that the proportion of patients presenting with myelitis was 19.3%, followed by ON (67.5%) and encephalitis (25.6%) (Akaishi et al., 2019). Of the 263 demyelinating episodes in 93 MOGAD patients recorded by Netravathi et al. (2020), myelopathy accounted for 22.8%, second only to ON (45.8%). Cobo-calvo’s case sequence showed that 42.9% of MOGAD patients had myelitis as the first symptom (Cobo-Calvo et al., 2017). Kitley et al. (2014) reported that compared with NMOSD patients, MOGAD patients had the highest initial symptoms of ON and myelitis (44 vs. 0%) occurring sequentially (within 1 month) (*p* = 0.005), and myelitis alone was present in 33% of patients.

#### Symptom Features

Myelitis in MOGAD patients is usually long extensive transverse myelitis (LETM) (≥ 3 vertebral lengths), which is very similar to NMOSD (Akaishi et al., 2019). The cervical and thoracic spinal cords are the most frequently involved parts, and the posterior medulla or posterior region of the cervical spinal cord may be involved (Zhong et al., 2019; Ciron et al., 2020). In Netravathi et al. (2020), 70.1% of MOGAD patients developed myelitis with long segmental lesions. Additionally, sagittal “H-pattern” lesions similar to those in patients with NMOSD are observed (Netravathi et al., 2020; Mariano et al., 2021). Cobo-calvo’s case sequence showed that 80% (8 of 10) of MOGAD patients who presented with myelitis had LETM (Cobo-Calvo et al., 2017). However, several MOGAD patients who present with recurrent short extensive myelitis have been reported (Spadaro et al., 2016; Breza et al., 2019; Dolbec et al., 2020; Zheng et al., 2021). Kitley’s results showed that the proportion of MOGAD patients with conus involvement was significantly higher than that of NMOSD patients (75 vs. 17%, *p* = 0.02) (Kitley et al., 2014).

#### Magnetic Resonance Imaging Features

Spinal MRI usually presents longitudinally extensive lesions (61.3 to 92.9%) or scattered short lesions (38.4 to 48%) as hyperintense in T2 sequences (Baumann et al., 2015; Jarius et al., 2016; Dubey et al., 2019; Mariano et al., 2019; Ciron et al., 2020). Lumbosacral spinal cord involvement is a characteristic manifestation of MOGAD, which is significantly higher than that in NMOSD and MS patients (Kitley et al., 2014; Sato et al., 2014). Carandini’s meta-analysis yielded similar results (OR = 3.47; 95% CI = 1.66–7.24; *p* = 0.0009) (Carandini et al., 2021). The incidence of lumbar medulla and conus involvement has been reported to be approximately 30.1-41% (Dubey et al., 2019; Ciron et al., 2020; Rempe et al., 2021). Contrast-enhanced MRI of spinal cord lesions usually shows scattered or patchy enhancement (Jarius et al., 2016; Dubey et al., 2019). Rarely, 10% of MOGAD patients presenting with myelitis may have a normal initial spinal MRI, but half of them have spinal lesions during follow-up (Sechi et al., 2021).

#### Outcome and Prognosis

The symptoms of myelitis include limb numbness and weakness, defecation and urine obstacles. Akaishi et al. (2019) used the multiple sclerosis severity score (MSSS) to evaluate the functional status of MOGAD patients after treatment, and the results showed no difference in the scores of patients with and without myelitis (0.83 ± 1.71 vs. 1.09 ± 1.98; *p* ≥ 0.10). This suggests that myelitis in MOGAD patients, when treated promptly, usually recovers well and does not leave permanent disability. The results of Kitley et al. (2014) showed that the median EDSS score of convalescing MOGAD patients was significantly lower (*p* = 0.01) and the median difference in the EDSS score was higher (*p* < 0.001) than those in NMOSD patients. Additionally, in 6 of 7 patients, spinal MRI at follow-up (median, 9.5 months; range, 5-17 months) showed complete disappearance of T2 hyperintense lesions with no residual spinal atrophy. Jarius et al. (2016) reported that 40% of MOGAD patients presenting with ON or myelitis had complete or near-complete recovery after plasma exchange therapy. However, it has also been reported that 50-80% of patients exhibit long-term disability (including decreased motor ability and bladder and bowel dysfunction) and that transverse myelitis as the onset symptom is a strong predictor (Jurynczyk et al., 2017). Jurynczyk’s study showed that half of MOGAD patients exhibited long-term disabilities, most often sphincter and erectile dysfunction, but vision and motor disabilities were rare (Jurynczyk et al., 2017). Netravathi et al. (2020) reported that 20.4% of MOGAD patients may exhibit long-term gait instability and lower limb weakness.

#### Relapse

Similarly, MOGAD patients presenting with myelitis may also experience recurrence. Additionally, it is not uncommon for MOGAD patients to present with recurrent myelitis of the same length. Interestingly, patients presenting with short extensive myelitis had a higher recurrence rate than patients with LETM (mean: 0.35 vs. 0.13) (Ciron et al., 2020). In a study of 70 MOGAD patients, Chen et al. reported a 49% recurrence of myelitis, second only to ON (96%) (Chen et al., 2020). Kunchok et al. (2021a) reported that of 14 MOGAD patients, only 1 presented with initial symptoms of myelitis, but 4 presented with recurrent symptoms.

### Acute Disseminated Encephalomyelitis

#### Epidemic

Acute disseminated encephalomyelitis is the most common clinical manifestation of MOGAD in children and is less common in adults than ON and myelitis (Reindl et al., 2013; Reindl and Rostasy, 2015; Hacohen et al., 2017; Jurynczyk et al., 2017; Netravathi et al., 2020; Vibha et al., 2021). The results of Ramanathan’s study of 33 children and 26 adults with MOGAD found that 20% of the population presented with ADEM (exclusively in children), second only to ON (54%) (Ramanathan et al., 2018). Kortvelyessy et al. (2017) reported two adult patients presenting with intracranial perivascular microlesions that were eventually found to be MOG-IgG positive. Brilot’s results showed that 40% of children with clinically isolated syndrome (CIS) or ADEM were positive for MOG-IgG (Brilot et al., 2009). Hennes et al. (2017) found that 57% of children with ADEM were positive for MOG-IgG. Lopez-Chiriboga’s results showed that 25 of 51 patients (49%) with onset-isolated ADEM were positive for MOG-IgG (Lopez-Chiriboga et al., 2018).

#### Symptom Features

Symptoms of infection or other diseases are usually observed in 70-80% of patients before onset. Even so, onset is usually sudden and progresses within a few days (Tenembaum et al., 2007; Paolilo et al., 2020). The common symptoms of MOGAD patients who present with ADEM include epilepsy, mild paralysis, ataxia, dysarthria and difficulty walking and altered sensorium, even dementia and confusion (Cobo-Calvo et al., 2018; Nagabushana et al., 2019; Bruijstens et al., 2020). Different symptoms appear according to the site and scope of the lesions involved. A small number of pediatric patients may require ICU admission (Ketelslegers et al., 2011). The disease is usually relieved in the weeks following the peak. In recent years, several MOGAD patients presented with bilateral frontal cortex involvement have been reported, described by some scholars as anti-myelin oligodendrocyte glycoprotein (MOG) antibody-associated bilateral medial frontal cerebral cortical encephalitis (BFCCE). The most common symptoms are headache and fever, but also include seizures, hemiplegia and fatigue (Fujimori et al., 2017, 2020; Cobo-Calvo et al., 2018).

#### Magnetic Resonance Imaging Features

Magnetic resonance imaging of MOGAD patients who present with ADEM typically shows cortical or subcortical hyperintense lesions on fluid-attenuated inversion recovery (FLAIR) sequences, with heterogeneous enhancement (scattered linear and nodular) post-gadolinium (Ogawa et al., 2017; Hamid et al., 2018; Leinert et al., 2020). Lesions appear in both the supratentorial and infratentorial regions and are usually paler, poorly demarcated and fewer in number (Lopez-Chiriboga et al., 2018; Akaishi et al., 2019). Notably, MOGAD lesions do not fulfill the criteria for MS or NMOSD and MRI features of MOGAD and NMOSD have been summarized (Table 2). Some reports have disclosed brainstem lesions, encephalopathy or cortical encephalitis (Cobo-Calvo et al., 2018). Cobo-calvo’s results show that thalamic and brainstem involvement are a unique sign of MOGAD. Additionally, abnormal lesions (usually bilateral) have been reported in 45% of MOGAD patients on their first cranial MRI (Cobo-Calvo et al., 2018). The results of Tenembaum et al. (2002) also indicated that bilateral thalamic lesions occurred in 63% of MOGAD patients who presented with ADEM. Kitley et al. (2014) reported that MOGAD patients had more deep gray matter and adjacent to the fourth ventricle lesions than NMOSD patients. Compared with NMOSD patients, cerebellar peduncle lesions were only seen in children with MOGAD (Fujimori et al., 2017). In MOGAD patients with BFCCE, the most characteristic radiographic appearance is bilateral frontal cortex Flair hyperintense lesion on MRI. The lesion is located in the area supplying the anterior cerebral artery (Fujimori et al., 2017). In one study, compared to healthy controls, decreased parallel diffusivity within white matter was observed in MOGAD patients, while NMOSD patients showed no significant difference. NMOSD patients showed decreased whole brain volumes and volumes of several deep gray matter structures, but MOGAD patients showed no significant difference in DTI analysis (Schmidt et al., 2020).

**TABLE 2:** Brain MRI features comparison between MOGAD and NMOSD.

	MOGAD	NMOSD
MRI features; Lopez-Chiriboga et al., 2018; Akaishi et al., 2019; Yang et al., 2020	paler, poorly demarcated	fluffy, confluent
	lager size	
	dispersed distribution	
Location; Cobo-Calvo et al., 2018; Kunchok et al., 2020; Rosenthal et al., 2020; Shor et al., 2021	normal and/or non-specific	normal and/or non-specific
	similar to ADEM	hypothalamic
	thalamic, brainstem	periaqueductal gray
	adjacent to the fourth ventricle	medulla oblongata and area postrema
	deep gray matter	adjacent to the
	white matter cortical(rare)	third ventricle
Enhancement; Yan et al., 2016; Fujimori et al., 2020; Salama et al., 2020	scattered linear and nodular enhancement	often with enhancement
Outcome; Kitley et al., 2014; Shor et al., 2021	Resolution	Resolution

*MOGAD, myelin oligodendrocyte glycoprotein-IgG-associated disorder; NMOSD, neuromyelitis optica spectrum disorder; MRI, magnetic resonance imaging.*

#### Outcome and Prognosis

Jurynczyk et al. (2017) reported that of 15 MOGAD patients who presented with ADEM, 6 (40%, three pediatric patients and three adult patients) exhibited long-term cognitive impairment. Pohl et al. (2016) and Deiva et al. (2020) reported similar results (Pohl et al., 2016; Deiva et al., 2020). Rossor et al. (2020) found that MOG-IgG positive children who presented with ADEM were more likely to develop post disease epilepsy than MOG-IgG negative children. Lopez-Chiriboga et al. (2018) followed 25 MOGAD patients who presented with isolated ADEM with a median follow-up EDSS score of 0 or 1.5 (children or adults, respectively) and reported similar results with or without recurrence. Kitley et al. (2014) followed up 6 MOGAD patients who had brain lesions on MRI at the time of the first symptom and re-examined cranial MRI at the time of symptom convalescence, all of them showed complete disappearance of the lesions.

#### Relapse

After timely immunotherapy (corticosteroids or plasma exchange), ADEM usually presents as a monophasic course and is mostly self-limited (Young et al., 2008). However, some patients can relapse and exhibit myelitis, ON and other symptoms (Jurynczyk et al., 2017). Cobo-Calvo’s results showed that adult MOGAD patients who presented with ADEM had a higher risk of recurrence than children (HR 1.63, 95% CI 0.99-2.69; *p* = 0.057) (Cobo-Calvo et al., 2021). Lopez-Chiriboga et al. (2018) studied the effect of persistent MOG-IgG on relapse in MOGAD patients. The results showed that 15 of the 17 patients with persistent MOG-IgG positivity had at least one recurrent event, while only 1 of the 8 patients with transient MOG-IgG positivity had a recurrence. Compared with that in ADEM patients who were double negative for MOG-IgG and AQP-4 antibody, the HR for relapse in the persistent MOG-IgG group was 3.1 (95% CI, 1.1-8.9; *P* = 0.04) in children and 5.5 (95% CI, 1.4-22.5; *P* = 0.02) in adults.

## Other

### Aseptic Meningitis

**FIGURE 1:**
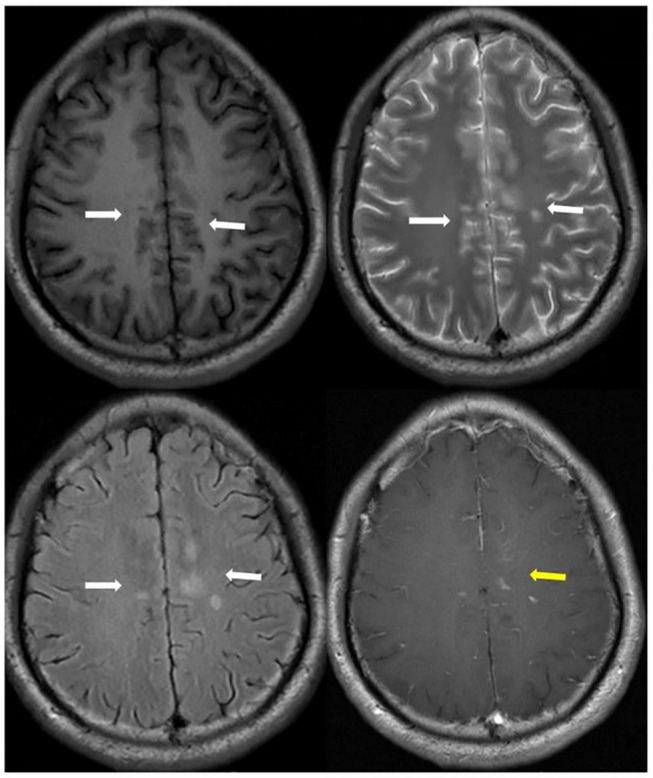
A patient presented MOG-Ab positive followed aseptic meningitis. Axial T1 (upper left), T2 (upper right), T2-Flair images (bottom left) showed long signal lesions on bilateral cingulate gyrus, right corpus callosum and frontal lobe, left semi-oval center (white arrowhead). T1-contrast weight images (bottom right) showed nodular enhancement (yellow arrowhead).

**FIGURE 2:**
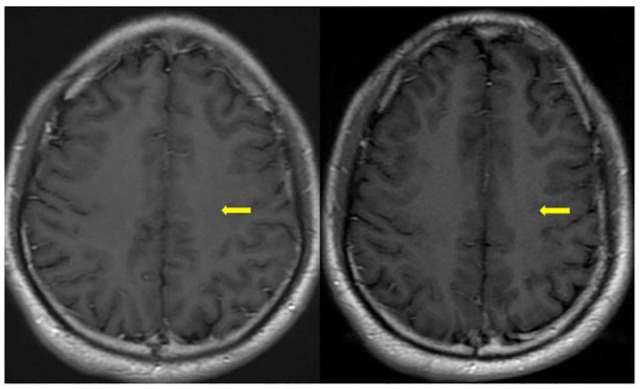
One month later (left) and three months later (right) axial T1-contrast weight images showed nodular enhancement disappeared (yellow arrowhead) after hormone therapy.

Currently, approximately 11 aseptic meningitis cases accompanied by MOGAD have been reported (Narayan et al., 2019; Suzuki et al., 2019; Leinert et al., 2020; Miller et al., 2020; Shi et al., 2020; Shen et al., 2021; Vibha et al., 2021; Zhang et al., 2021). Headache, fever, and ON were the most common symptoms, with mildly higher CSF cells, except in one case (1335/dL) (Miller et al., 2020). Brain imaging showed abnormal signals in various locations. Blood brain barrier disruption induced by preceding infection plays a role in permitted MOG-IgG access to the CNS and results in demyelination (Nagabushana et al., 2019; Suzuki et al., 2019). Animal experiments demonstrated that aseptic meningitis was induced in CD28-deficient C57BL/6 mice after immunization with MOG (Perrin et al., 1999). Nagabushana et al. (2019) reported that 45% of MOGAD patients have infectious or flu-like symptoms prior to onset. CSF pleocytosis occurs in 44–85% of MOGAD patients (Jurynczyk et al., 2017; Mariotto et al., 2017; Cobo-Calvo et al., 2018; Ramanathan et al., 2018). Therefore, patients initially diagnosed with meningitis in the clinic should pay attention to exclude MOGAD even if there are no symptoms when intracranial abnormal lesions appear (Figures 1, 2).

### FLAIR-Hyperintense Lesions in Anti-MOG Antibody-Associated Cerebral Cortical Encephalitis With Seizures (FLAMCES)

FLAMCES is a recently reported subtype of MOGAD that is mainly characterized by hyperintense lesions on MRI FLAIR sequences, cortical encephalitis, and seizures. Approximately 21 cases have now been reported (Budhram et al., 2019; Mirian et al., 2021; Wang et al., 2021). According to Wang’s review, the age of onset was younger (26.8 years on average), with a larger proportion of males (76.2%). Common symptoms of FLAMCES include headache, fever and cortical symptoms (aphasia, hemiparesis, hemianopsia, memory defects, and psychiatric symptoms). Epilepsy consists of many types of seizures, but a persistent epileptic state rarely occurs. Of 21 patients, seven had recurrent events during the follow-up period, including ON, myelitis, brainstem syndrome, and ADEM. The majority of patients (17/21) presented with unilateral lesions, and of the 11 patients with enhanced MRI data, 7 presented with meningeal enhancement. All the patients received glucocorticoid therapy and showed recovery of symptoms and lesions. Some patients (9/21) were treated with antiepileptic drugs, and no unprovoked seizures were reported (Wang et al., 2021). Because seizures are thought to be caused by encephalitis, long-term antiepileptic drugs are not recommended.

### Anti-NAMDA Encephalitis

Encephalitis is a common clinical symptom in MOGAD patients, and the NAMDA antibody has been found in some MOGAD patients in recent years (Wang et al., 2019; Amano et al., 2020; Fujimori et al., 2021). Fujimori et al. (2021) reported a case of a patient presenting with cortical symptoms such as headache, fever, seizure, and cognitive impairment. The patient was initially diagnosed with anti-NMADA encephalitis. Later, due to two recurrences, the patient’s cerebrospinal fluid at onset was tested again, and MOG-IgG was found to be positive. Amano et al. (2020) also reported a similar case. The results of Wang et al. indicated that among 87 MOGAD patients, 18 patients (20.7%) could be diagnosed with encephalitis after evaluation of clinical symptoms and imaging. Five of these patients tested positive for NMADA antibody during the encephalitis course (Wang et al., 2019). Kunchok et al. (2021a) studied patients in which MOG-IgG coexisted with antibodies associated with autoimmune encephalitis. A total of 376 MOG-IgG positive patients were tested. The results showed that among 255 blood samples (113 adults and 142 children), one child was associated with NMADA antibody, one child with CASPR2 antibody, one adult with LGI1 antibody, and one adult with GABAA antibody. Among 266 cerebrospinal fluid samples, seven children and seven adults had NMADA antibody, and one adult had GABAA antibody. NMADA antibody was the most common combination antibody, and patients who were double positive for NMADA and MOG-IgG were more likely to experience encephalopathy (*p* = 0.001), seizures (*P* = 0.045), and leptomeningeal enhancement (*p* = 0.045). Anti-NMADA encephalitis may be related to herpesvirus infection, and MOGAD can also be induced by infection (Nakamura et al., 2017; Khan et al., 2021; Mbonde et al., 2021; Peters et al., 2021). This may be one mechanism of MOG and NMADA antibody double positivity (Reindl et al., 2013; Armangue et al., 2015). Generally, after immunotherapy, most patients recover completely, such as those with ON or myelitis, and the degree of recovery is not significantly different from that of patients with a single positive MOG-IgG (Wang et al., 2019; Amano et al., 2020; Fujimori et al., 2021; Kunchok et al., 2021a).

### Peripheral Nervous System Involvement

The results of Rinaldi’s study found that of 271 MOGAD patients, 19 had combined Peripheral Nervous System (PNS), including acute inflammatory polyneuropathy (*n* = 1), myeloradiculitis (*n* = 3), multifocal motor neuropathy (*n* = 1), brachial neuritis (*n* = 2), migrant sensory neuritis (*n* = 3), and paresthesia and/or radicular pain (*n* = 10) (Rinaldi et al., 2021). Of the 15 patients (15 of 19) who underwent immunotherapy, 3 recovered completely, 9 partially, and 3 showed no significant improvement. Five patients also had PNS recurrence. Nakamura et al. (2021) reported a case of a MOGAD patient presenting with peripheral neuropathy with multiple spinal and intracranial lesions and ventral and dorsal nerve root thickening. She exhibited recurrence several times after intravenous methylprednisolone (IVMP) treatment, which was similar to chronic inflammatory demyelinating peripheral neuropathy (CIDP). The patient was consistently positive for MOG-IgG at the last follow-up (titer 1:256). MOGAD may affect nerve roots or cause a peripheral immune response, which may be the underlying mechanism.

### Myelin Oligodendrocyte Glycoprotein-IgG-Associated Disorder Associated With Other Diseases

Myelin oligodendrocyte glycoprotein-IgG-associated disorder associated with tumors has rarely been reported Wildemann et al. (2021) reported a right ovarian teratoma case that was surgically removed followed by recurrent ON, in which the MOG-IgG was positive (titer 1:320). Symptoms were relieved after IVMP. MOG-IgG was negative 2 and 4 months after the second attack. Histological results showed the presence of nerve tissue expressing MOG protein and immune cell infiltration in the teratoma. This may be a possible paraneoplastic mechanism in MOGAD patients, but more evidence is needed.

Fujimori et al. (2019) reported two cases of MOG-IgG positivity in patients with clinical manifestations similar to neurological Behcet’s disease. The first patient presented with focal motor seizures, and cerebral MRI showed multiple cortical and subcortical hypersignal lesions on both T2 and FLAIR sequences. Acne was observed on the skin. Behcet’s disease was considered. In 2018, serum was found to be positive for MOG-IgG (titer 1:256). Another patient, who also presented with neurological Behcet’s disease symptoms, was found to be MOG-IgG positive in remission (1:128). The specific mechanisms are unknown, but the possibility of MOG-IgG positivity should be considered in patients with neuro-Behcet’s disease with uncommon brain stem involvement, such as cerebellar peduncles (Fujimori et al., 2019).

Chen et al. (2017) reported on a child who presented with fatigue, fever, and vomiting and developed ataxia and dysarthria accompanied by multiple subcortical white matter lesions at T2 and FLAIR sequences. She was diagnosed with Hashimoto’s thyroiditis two years prior. Hashimoto’s encephalopathy was the preliminary diagnosis. The symptoms gradually disappeared after IVMP plus long-term oral glucocorticoid treatment. Later, the retained cerebrospinal fluid test was positive for MOG-IgG. MOGAD may coexist with other systemic autoimmune diseases. It remains unknown whether MOG-IgG has a certain effect and needs further confirmation.

Nakamura et al. (2017) reported a case of bilateral ON following herpesvirus infection, and the patient was later found to be serum positive for MOG-IgG. Khan et al. (2021) and Peters et al. (2021) reported a case of MOGAD following COVID-19 infection. Mbonde et al. (2021) reported a well-controlled human immunodeficiency virus (HIV)-positive patient with bilateral parotid swelling and myelitis who was found to be double positive for MOG-IgG and mumps-IgG. Symptoms improved after glucocorticoid therapy without recurrence (1.5 years follow-up). The likely mechanism is that viral infection triggers an immune response, forming MOG-IgG.

## Limits and Future Directions

Most of the studies discussed in this paper are retrospective studies or case series reports, and data from large prospective studies are still scarce. Future trials are needed to obtain more information about the clinical manifestations of MOGAD. At present, there is still a lack of unified imaging characteristics, diagnostic criteria and treatment methods for MOGAD. A large number of clinical imaging data and various indicators should be collected to help clinicians make rapid and accurate diagnosis and develop treatment strategies. There is still a lack of long-term follow-up data to help judge the outcome, which should be improved as soon as possible.

## Conclusion

Myelin oligodendrocyte glycoprotein-IgG-associated disorder is currently recognized as an independent demyelinating disease of the CNS. The onset age is variable, with a median age of approximately 40 years, and MOGAD can occur in both adults and children. There is no significant sex difference in MOGAD. The main symptoms include ON, myelitis and ADEM, but many atypical symptoms have been reported. Usually, the prognosis is satisfactory, with complete or partial recovery after immunotherapy (Table 3). Some patients may have recurrence, and long-term oral glucocorticoids may reduce the probability of recurrence.

**TABLE 3:** Recommendation on treatment in MOGAD patients.

	Acute attack	Maintenance treatment
IVMP; Jarius et al., 2016	first-line	
	large dose, ladder reduction	
Oral steroids	first-line	first-line
	slow tapering	min 3 months
	6-12months	
IVIG	adjuvant therapy	adjuvant therapy
	5 days	monthly
Plasmapheresis; Spadaro et al., 2016	after IVMP and IVIG	
	5-7 times	
Rituximab; Jarius et al., 2016		combination with oral steroids
AZA; Jarius et al., 2016; Zhou et al., 2017		alone or combination with oral steroids
		long-term maintenance at low doses
MMF		combination with oral steroids

*MOGAD, myelin oligodendrocyte glycoprotein-IgG-associated disorder; IVMP, intravenous methylprednisolone; IVIG, intravenous immunoglobulins; AZA, azathioprine; MMF, mycophenolate mofetil.*

## Author Contributions

YL, XL, JW, and CP contributed to the study conception, design, and data analysis. YL wrote the manuscript. CP and ZT reviewed and edited the manuscript by language polishing. All authors have participated in the work, and have reviewed and agreed with the content of the article.

## Conflict of Interest

The authors declare that the research was conducted in the absence of any commercial or financial relationships that could be construed as a potential conflict of interest.

## Publisher’s Note

All claims expressed in this article are solely those of the authors and do not necessarily represent those of their affiliated organizations, or those of the publisher, the editors and the reviewers. Any product that may be evaluated in this article, or claim that may be made by its manufacturer, is not guaranteed or endorsed by the publisher.
